# Effects of different exercises on motor and non-motor abilities in patients with Parkinson disease—a network meta-analysis of randomized controlled trials

**DOI:** 10.3389/fphys.2026.1809614

**Published:** 2026-05-01

**Authors:** Jianfeng Tang, Xinhong Liu, Jianqiang Guan, Sitao Li, Zenghui Xing

**Affiliations:** 1 School of Physical Education, Northeast Normal University, Changchun, China; 2 Changchun University of Science and Technology, Changchun, China; 3 Department of Physical Education, Northeastern University at Qinhuangdao, Qinhuangdao, China; 4 Copper Springs Behavioral Health Hospital, Avondale, AZ, United States

**Keywords:** aerobic exercise, cognitive level, goal-directed training, motor skills, non-motor skills, Parkinson’s disease, traditional exercise

## Abstract

**Objective:**

To systematically investigate and evaluate the effects of different exercise (TT: treadmill training; ST: sensorimotor training; NW: Nordic walking; CT: cycling training; WT: walking training; DT: dance training; VRT: VR training; RT: resistance training; AQT: aquatic training; BOX: boxing training; Qigong and Yoga) modalities on both motor and non-motor abilities in patients with Parkinson’s disease.

**Methods:**

Randomized controlled trials (RCTs) concerning the impact of various exercise modes on motor and non-motor abilities in Parkinson’s patients were identified by searching the PubMed, Web of Science, Cochrane library, CINAHL and CNKI databases. The search period spanned from the inception of each database to December 2025. The methodological quality of the included studies was assessed using the Cochrane Risk of Bias tool.

**Results:**

A total of 67 RCTs involving 2642 patients were included. Network meta-analysis results indicated that WT most effectively improved UPDRS-III (SUCRA=92.5%); DT was most effective in improving TUG (SUCRA=89.9%) and 6MWT (SUCRA=73.3%); NW best improved BBS (SUCRA=91.1%) and MoCA (SUCRA=91.1%). AQT best improved PDQ-39 (SUCRA=88.5%).

**Conclusion:**

Current evidence suggests that exercise involving walking and dance, which stimulates neural regulation, can help improve motor function and cognitive abilities in people with Parkinson’s disease. Patients can choose exercises based on their current fitness level.

**Systematic review registration:**

https://osf.io/7x5kc/.

## 1 Introduction

The proportion of the global elderly population continues to rise, accompanied by an increasing burden of age-related diseases. Notably, the incidence of Parkinson’s disease (PD) shows a significant positive correlation with age (Schootemeijer et al., 2020), and its steadily rising prevalence poses a major challenge to public health systems worldwide. PD occurs more frequently in middle-aged and elderly populations, with its typical clinical manifestations including progressive cognitive impairment and memory decline. From a pathological perspective, Parkinson’s disease is characterized by the progressive degeneration and death of dopaminergic neurons in the substantia nigra pars compacta of the midbrain, accompanied by the deposition of Lewy bodies composed of alpha-synuclein within these neurons (Schootemeijer et al., 2020). This leads to characteristic motor impairments, including bradykinesia, resting tremor, rigidity, and postural instability. As the disease progresses, pathological involvement extends to the cerebral cortex and other neurotransmitter systems, subsequently triggering a range of non-motor symptoms such as comprehensive impairment in cognitive function and executive function (Zhang et al., 2018). Based on these characteristics, assessments of cognitive function, emotional state, and motor performance have become crucial indicators for PD clinical interventions.

Since there is currently no cure for Parkinson’s disease, and drug treatments carry a range of side effects (Duncker and Bache, 2008), exercise interventions have gained increasing attention in recent years as a complementary approach to PD management. Numerous studies indicate that regular physical activity can reduce the risk of developing PD and positively impact both motor and non-motor symptoms in patients. These benefits include improved motor function, reduced risk of falls, enhanced cognitive abilities and increased overall quality of life (Ernst et al., 2023; Lei et al., 2025).

However, the improvement effects achieved by different exercise programs may vary. Most current meta-analyses focus on specific exercise types (Zhou et al., 2022) and fail to systematically evaluate all exercise modalities. Although the American Academy of Neurology (AAN) has highlighted the benefits of Tai Chi in enhancing patients’ balance (Gladfelter, 2011), and the Chinese Association of Rehabilitation Medicine recommends traditional exercises (such as Tai Chi and Baduanjin) as exercise interventions for Parkinson’s disease while increasing cultural adaptability, their comparative metrics primarily emphasize patients’ motor performance. (Pang et al., 2022) For Parkinson’s patients, quality of life and cognitive function are equally critical, as non-motor symptoms can directly erode psychological wellbeing and social engagement (Malkki, 2013). To address these gaps, this study employs a network meta-analysis to systematically compare the effects of different exercise modalities on motor performance, cognition and quality of life in PD patients. This review aims to provide evidence-based recommendations for personalized and comprehensive exercise intervention strategies.

## 2 Materials and methods

The study was conducted, and the manuscript was prepared in accordance with the Preferred Reporting Items for Systematic Reviews and Meta-Analyses (PRISMA) guidelines (Page et al., 2021). The study was registered with Open Science Framework, https://osf.io/7x5kc/.

### 2.1 Search strategy and study selection

**Figure 1:**
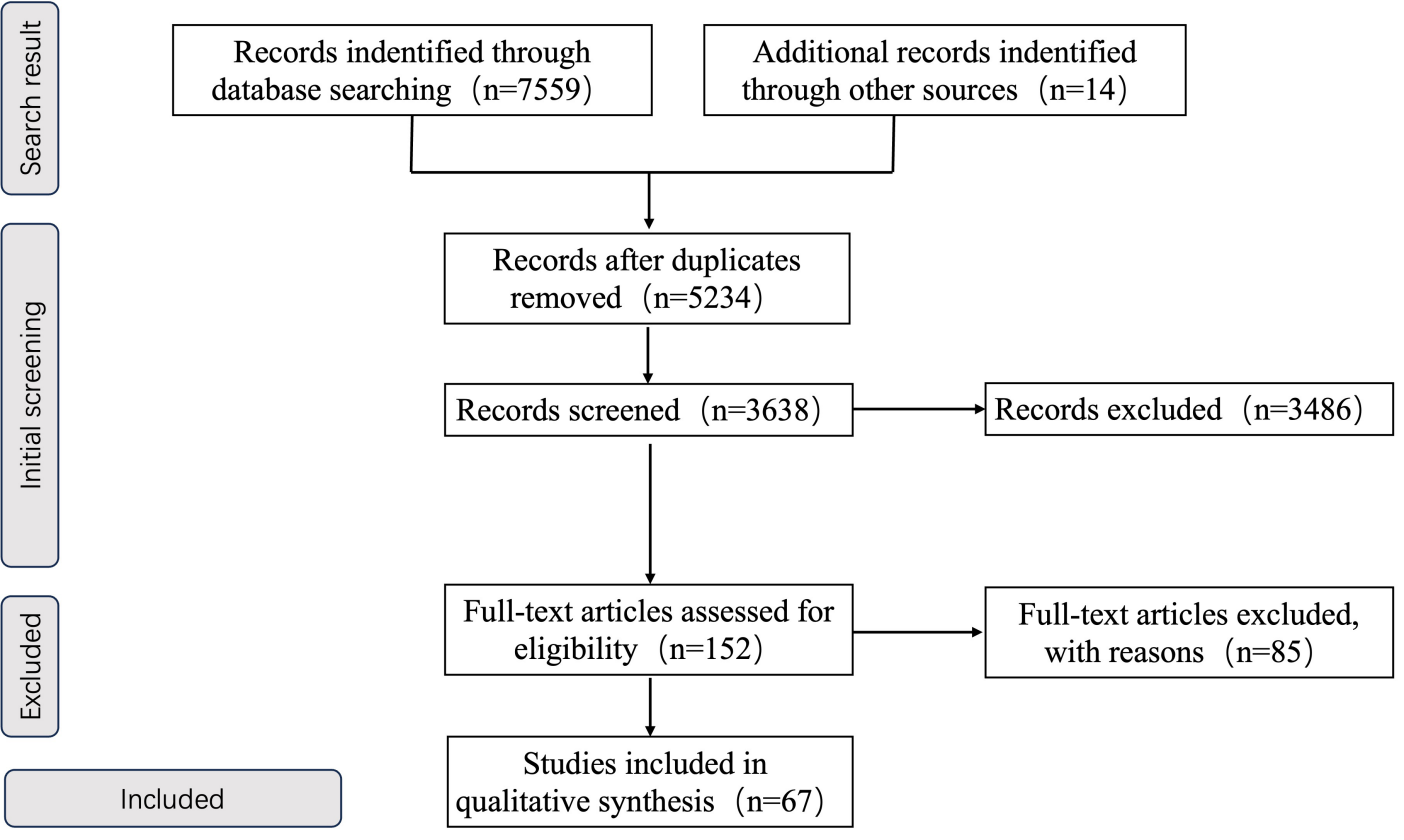
Flow chart illustrating the different phases of the search and study selection.

We conducted a systematic literature search across multiple databases including PubMed, Web of Science, Cochrane library, CINAHL and CNKI. The search scope covered the period from each database’s inception to December 2025. The search strategy combined Medical Subject Headings (MeSH) terms with free-text keywords, finalized after multiple preliminary tests. Additionally, manual searches and review of reference lists from included studies were employed to identify further relevant research. Search terms included “Parkinson’s disease,” “exercise,” “movement,” “athletic ability,” “Balance,” “cognition,” “depression,” and “quality of life,” combined using Boolean operators. Two reviewers (JF and XH) independently screened titles and abstracts to identify potentially relevant articles, followed by full-text review of selected documents. Additional relevant articles were identified by consulting the reference lists of articles retrieved from the initial database search. **Figure 1** illustrates the flowchart describing the literature selection process.

Search results were imported into Zotero software (version 6.0.30) to remove duplicates. Two researchers conducted an initial assessment of the titles and abstracts of the retrieved literature, then read the full texts for further screening. When disagreements arose during article screening, a third researcher made the final decision.

### 2.2 Inclusion and exclusion criteria

Inclusion criteria were established based on the PICO framework: (1) All participants had been diagnosed with Parkinson’s disease by a neurologist, based on the clinical diagnostic criteria established by the Movement Disorder Society (MDS) (Postuma et al., 2015); (2) The study was a randomized controlled trial (RCT); (3) The intervention was exercise-based; (4) Outcomes: Motor performance assessed via Unified Parkinson’s Disease Rating Scale (UPDRS III), Timed Up and Go Test (TUG), Berg Balance Scale (BBS), and 6-Minute Walk Test (6MWT); non-motor function evaluated using Parkinson’s Disease Quality of Life-39 (PDQ-39) and Montreal Cognitive Assessment (MoCA).

Exclusion criteria were as follows: (1) conference abstracts, case reports, commentaries, reviews, and animal studies; (2) outcome measures not meeting inclusion criteria; (3) incomplete data where mean and standard deviation could not be obtained from the literature or author communication.

### 2.3 Data extraction

Using seven standardized data extraction forms, record the following data from included studies under the following headings: (1) Authors, (2) Publication year, (3) Country, (4) Study period, (5) Sample size, (6) Mean age, (7) Exercise intervention details. (8) Outcomes: Motor performance assessed via Unified Parkinson’s Disease Rating Scale (UPDRS III), Timed Up and Go Test (TUG), Berg Balance Scale (BBS), and 6-Minute Walk Test (6MWT); non-motor function evaluated using Parkinson’s Disease Quality of Life-39 (PDQ-39) and Montreal Cognitive Assessment (MoCA).

### 2.4 Risk of bias of individual studies

The risk of bias in the included studies was assessed using the Cochrane risk of bias tool. Two researchers independently evaluated seven domains: random sequence generation (selection bias), allocation concealment (selection bias), blinding of participants and personnel (performance bias), blinding of outcome assessment (detection bias), incomplete outcome data (attrition bias), and selective reporting (reporting bias). Each study was categorized as “high risk,” “low risk,” or “uncertain risk.” Disagreements were resolved through discussion or consensus among a third researcher. Studies were classified into three groups: high risk (five or more risk factors), moderate risk (three to four risk factors), and low risk (two or fewer risk factors) (Higgins et al., 2011).

### 2.5 Data analysis

Using R software (RStudio V4.19, Boston, Massachusetts, USA), effect size pooling and comparisons were conducted within a Bayesian framework employing the Markov Chain Monte Carlo (MCMC) method, based on the PRISMA network meta-analysis guidelines (Shamseer et al., 2015). The random-effects NMA model was fitted using functions from the MCMCpack package. Consistency in direct and indirect comparisons was quantified and visualized using the node method, with consistency established when P-values > 0.05 (Salanti et al., 2011).

Network diagrams depicting different exercise interventions were generated and described using R software. Within the generated network diagrams, each node represented a distinct exercise intervention and control condition, while connecting lines indicated direct head-to-head comparisons between interventions. Node size and link width are proportional to the number of studies. Based on MCMC posterior samples, the probability of each intervention occupying different ranking positions (from best to worst) is calculated. The cumulative area under the ranking curve (SUCRA) value is then computed. SUCRA ranges from 0% to 100%, with higher values indicating a higher-ranked intervention (better efficacy). Although SUCRA values can be effectively converted into percentages representing the effectiveness or acceptability of exercise interventions, such scores should be interpreted cautiously unless clinically meaningful differences exist between interventions. To detect bias arising from small-scale studies, network funnel plots were generated and visually inspected according to symmetry criteria.

## 3 Results

See **Table 1**. This study included 67 RCT publications. A total of 2642 patients from 19 countries were enrolled. 59 articles reported patients’ Hoehn-Yahr staging. The vast majority of articles were published within the last decade, with 14 articles published within the last five years.

**Table 1:** Characteristics of the studies included in the meta-analysis.

First author, year	country	Intervention-a	Intervention-b	Control	Sample size	years	Hoehn-Yahr	Outcome
(Altmann et al., 2016)	French	treadmill training 16 weeks Freq:three times a week Duration: 20-45min	BLT(balance training)	CON	11 or 9/10	63.3 ± 7.3	1-3	①
(Sage and Almeida, 2009)	USA	treadmill training 12 weeks Freq:three times a week Duration: 20min	–	CON	13/15	64.61±9.68	1-3	①③
(Nadeau et al., 2014)	Canada	treadmill training 3 months Freq:three times a week Duration: 60min	–	CON	30/34	64.0 ± 6.6	1-2	②
(Picelli et al., 2016)	Italy	treadmill training 4 weeks Freq:three times a week Duration: 45min	–	CON	9/8	71.2 ± 9.2	3	②⑤
(Carvalho et al., 2015)	USA	treadmill training 12 weeks Freq:two times a week Duration: 30min	resistance training 12 weeks Freq:two times a week Duration: 30min	BLT(balance training)	5/8/9	64.4±11.7	1-3	①
(Canning et al., 2012)	Australia	treadmill training 6 weeks Freq:four times a week Duration: 30-40min	–	CON	10/10	61.8±7.9	1-2	①②⑥
(Arcolin et al., 2016)	Italy	treadmill training 3 weeks Freq:five times a week Duration: 60min	cycling training 3 weeks Freq:five times a week Duration: 60min	–	18/13	68.32±8.38	1.5-3	①②③
(Bello et al., 2013)	Spain	walking training 5 weeks Freq:three times a week 10m	treadmill training 5 weeks Freq:three times a week 10m	–	11/11	58.75±10.17	1-3	①③
(Mitarnun et al., 2022)	Thailand	walking training 12 weeks Freq:three times a week Duration: 30min	–	CON	17/16	61.3 ± 9.1	1-3	①③
(Cugusi et al., 2015)	Italy	Nordic Walking 12 weeks Freq:two times a week Duration: 60min	–	CON	10/10	67.3 ± 7.8	1-3	①②③④
(Granziera et al., 2020)	Italy	Nordic Walking 8 weeks Freq:two times a week Duration: 75min	walking training 8 weeks Freq:two times a week Duration: 75min	–	18/18	68.5 ± 8.3	2-3	①③⑥
(Bang and Shin, 2017)	Korea	Nordic Walking 5 weeks Freq:five times a week Duration: 60min	treadmill training 5 weeks Freq:five times a week Duration: 60min	–	10/10	59.45±7.02	1-3	①③④
(Sacheli et al., 2019)	Canada	cycling training 12 weeks Freq:three times a week Duration: 60min	–	CON	20/15	66.76 ± 5.98	1-3	①③⑤
(Harper et al., 2019)	America	cycling training 12 weeks Freq:three times a week Duration: 60min	–	CON	20/15	65.05 ± 9.13	–	⑤
(Qutubuddin et al., 2013)	USA	cycling training 8 weeks Freq:two times a week Duration: 30min	–	CON	13/10	–	–	①④⑥
(Ferraz et al., 2018)	Brazil	cycling training 10 activities Duration: 30min	–	CON	25/22	63.2 ± 9.9	2-3	②⑥
(Hashimoto et al., 2015)	Japan	Dance training 12 weeks Freq:one time a week Duration: 60min	–	CON	15/14	67.9 ± 7.0	1-4	③④
(Hackney and Earhart, 2009)	USA	Dance training-Tango 13 weeks Freq:two times a week Duration: 60min	–	CON	14/17	67.27±2.4	1-4	②③④
(Solla et al., 2019)	Italy	Dance training-Tango 12 weeks Duration: 90min	–	CON	10/10	67.4 ± 6.1	≤3	①②③④
(Rios Romenets et al., 2015)	Canada	Dance training-tango 12 weeks Freq:two times a week Duration: 60min	–	CON	18/15	64.3 ± 8.1	1-3	①③⑤⑥
(Michels et al., 2018)	USA	Dance training-Dance to the rhythm of music 10 weeks Duration: 60min	–	CON	7/6	69.2 ± 8.7	2-2.5	①③④⑤⑥
(Volpe et al., 2013)	Italy	Dance training-Irish set dancing 24 weeks Freq:one time a week Duration: 90min	–	CON	12/12	63.3±5.11	1-2.5	①④⑥
(Duncan and Earhart, 2012)	USA	Dance training-community-based dancing 12 weeks Freq:two times a week Duration: 60min	–	CON	26/26	69.15±1.7	1-4	①
(Tollár et al., 2018)	Holland	VR training 3 weeks Freq:five times a week Duration:60 min	–	CON	35/20	67.3 ± 3.4	–	①③⑥
(Tollár et al., 2019)	Holland	VR training 5 weeks Freq:five times a week Duration:60 min	Cycling training 5 weeks Freq:five times a week Duration:60 min	CON	25/24	67.5±4.28	2-3	②④⑥
(Lee et al., 2015)	Korea	VR training 6 weeks Freq:two times a week Duration: 45min	–	CON	10/10	69.25±3.15	–	④
(Santos et al., 2019)	Brazil	VR training 8 weeks Freq:two times a week Duration: 50min	resistance training 8 weeks Freq:two times a week Duration: 50min	–	13/14	63.15±8.64	1-3	③④⑥
(van der Kolk et al., 2018)	Netherlands	VR training 6 months Freq:three times a week Duration: 30min	–	CON	15/22	30-75	1-2	①③⑥
(Song et al., 2018)	Australia	VR training 12 weeks Freq:three times a week Duration: Minimum 15 min	–	CON	31/30	66.52±7.10	1-3	③⑤
(Pazzaglia et al., 2020)	Italy	VR training 6 weeks Freq:three times a week Duration: 40min	–	CON	25/26	72 ± 7	1-3	④
(Yan et al., 2024)	China	resistance training 12 weeks Freq:three times a week Duration: 60min	–	CON	23/23	70.5 ± 5.4	≤3	①⑤⑥
(Amano et al., 2013)	USA	Qigong-Tai Chi 16 weeks Freq:two times a week Duration: 60min	–	CON	15/9	66±9.52	1-3	①
(Choi, 2016)	Korea	Qigong-Tai Chi 12 weeks Freq:two times a week Duration: 60min	–	CON	11/9	62.94±7.46	1-2	②③
(Zhu et al., 2020)	China	Qigong-Tai Chi 12 weeks Freq:two times a week Duration: 40~50 minutes	–	CON	19/22	68.53±1.90	2 ±2.2	①④⑤⑥
(Xiao et al., 2016)	China	Qigong-baduanjin 6 months Freq:four times a week Duration: 60min	–	CON	35/33	67.8±9.4	1-4	①②③④
(Wang Xiangyu et al., 2021)	China	Qigong-baduanjin 6 weeks Freq:seven times a week Duration: 60min	–	BLT(balance training)	27/24	65.52 ± 7.29	1-4	①
(Li et al., 2012)	USA	Qigong-Tai Chi 24 weeks Freq:two times a week Duration: 60min	resistance training 24 weeks Freq:two times a week Duration: 60min	BLT(balance training)	65/65	68.11±8.85	1-4	①③
(Khuzema et al., 2020)	India	Qigong-Tai Chi 8 weeks Freq:five times a week Duration: 30-40min	Yoga 8 weeks Freq:five times a week Duration: 30-40min	CON	9/9	70.06±5.4	2.5-3	③④
(Nocera et al., 2013)	USA	Qigong-Tai Chi 16 weeks Freq:three times a week Duration: 60min	–	CON	15/6	66 ± 11	1-3	⑥
(Vergara-Diaz et al., 2018)	USA	Qigong-Tai Chi 12 weeks Freq:two times a week Duration: 60min	–	CON	16/16	63.85±6.32	1-2	①③⑥
(Volpe et al., 2017)	Italy	Aquatic training 8 weeks Freq:five times a week Duration: 60min	–	BLT(balance training)	15/15	70.3±7.67	1-3	①③④⑥
(Kurt et al., 2018)	Turkey	Aquatic training 5 weeks Freq:five times a week Duration: 60min	–	BLT(balance training)	20/20	63.02±6.91	2-3	①③④⑥
(Volpe et al., 2014)	Italy	Aquatic training 8 weeks Freq:five times a week Duration: 60min	–	BLT(balance training)	17/17	68±7.52	2.5–3	①③④⑥
(vivas et al., 2011)	Spain	Aquatic training 4 weeks Freq:two times a week Duration: 45min	–	BLT(balance training)	12/12	67±5.59	1-3	④
(Carroll et al., 2017)	Italy	Aquatic training 6 weeks Freq:two times a week Duration: 45min	–	CON	10/8	71.5±4.67	1-3	①⑥
(Son and Choi, 2018)	Korea	resistance training 8 weeks Freq:one time a week Duration: 120 min	–	CON	33/30	66.5 ± 5.7	1-3	⑤
(Schlenstedt et al., 2015)	Germany	resistance training 7 weeks Freq:two times a week Duration: 60min	–	BLT(balance training)	17/15	75.7 ± 5.5	1-3	①③
(Santos et al., 2017)	spain	resistance training 8 weeks Freq:two times a week Duration: 60-70min	–	CON	13/15	73.6±15.86	1-3	①⑥
(Paul et al., 2014)	Australia	resistance training 12 weeks Freq:two times a week Duration: 45min	–	CON	20/20	66.3±6.73	–	③
(Ni et al., 2016b)	USA	resistance training 12 weeks Freq:three times a week Duration: 3 circuits of 10e12 repetitions on each machine	–	CON	10/14	73.3±7.45	1-3	⑥
(Goodwin et al., 2011)	UK	resistance training 10 weeks Freq:two times a week Duration: 50min	–	CON	61/63	72.0 ± 8.6	1-4	③④
(Ferreira et al., 2018)	Brazil	resistance training 6 month Freq:two times a week Duration:30-40min	–	CON	18/17	65.9±7.95	1-3	①⑥
(Dibble et al., 2009)	USA	resistance training 12 weeks Freq:three times a week Duration: 45-60min	–	CON	10/10	65.7±9.9	2-3	①③⑥
(Dibble et al., 2015)	USA	resistance training 12 weeks Freq:two times a week Duration: 50min	–	BLT(balance training)	20/21	68.4±11.95	–	①②
(Demonceau et al., 2017)	Belgium	resistance training 12 weeks Freq:two times a week Duration: 60-90min	cycling training 12 weeks Freq:two times a week Duration:	CON	20/17/15	66±9	2-3	③⑥
(Corcos et al., 2013)	USA	resistance training 24 months Freq:two times a week	–	BLT(balance training)	24/24	59.0 ± 4.6	–	①⑥
(Vieira de Moraes Filho et al., 2020)	Brazil	resistance training 9 weeks Freq:two times a week Duration: 50-60min	–	CON	25/15	64.6±5.5	1-3	①③
(Ni et al., 2016a)	USA	Yoga 12 weeks Freq:two times a week Duration: 60min	–	CON	13/10	71.2 ± 6.5	1-3	⑥
(Kwok et al., 2019)	China	Yoga 8 weeks Freq:one time a week Duration: 90min	resistance training 8 weeks Freq:one time a week Duration: 90min	–	71/67	63.6 ± 8.7	1-3	①③
(Cheung et al., 2018)	USA	Yoga 12 weeks Freq:two times a week Duration: 60min	–	CON	10/10	64.65±7.66	1-3	①⑤
(Combs et al., 2013)	USA	boxing training group 12 weeks 24–36 sessions Duration: 60min	resistance training 12 weeks 24–36 sessions Duration: 60min	–	14/17	68.0 ± 31.0	2.0 ± 3.0	②③④⑥
(Schenkman et al., 2012)	USA	sensorimotor training 4 months Freq:three times a week Duration: 50min	treadmill training 4 months Freq:three times a week Duration: 50min	CON	39/41/41	64.5 ± 10.0	1-3	①⑥
(Tanaka et al., 2009)	Brazil	sensorimotor training 24 weeks Freq:three times a week Duration: 60min	–	CON	10/10	65.4 ± 7.23	1-3	①
(Hubble et al., 2018)	Australia	sensorimotor training 12 weeks Freq:one time a week Duration: 90min	–	CON	11/11	65.4 ± 5.7	1-3	①③⑥
(Moratelli et al., 2025)	Denmark	sensorimotor training 12 weeks Freq:two times a week Duration: 60min	Yoga 12 weeks Freq:two times a week Duration: 60min	CON	11 or 8/9	63.3 ± 8.9	–	⑤
(Cholewa et al., 2013)	Poland	sensorimotor training 12 weeks Freq:two times a week Duration: 60min	–	CON	40/30	70.2 ± 5.75	3	①⑥
(Acarer et al., 2015)	Turkey	sensorimotor training 8 weeks Freq:two times a day Duration: 30-40min	–	CON	29/11	65.08 ± 8.09	2-3	①③④⑥

Outcome measures include: ①UPDRS-III ②6MWT ③TUG test ④BBS ⑤MoCA ⑥depression scale ⑦PDQ-39; “—” indicates not reported.

### 3.1 Risk bias assessment

**Figure 2:**
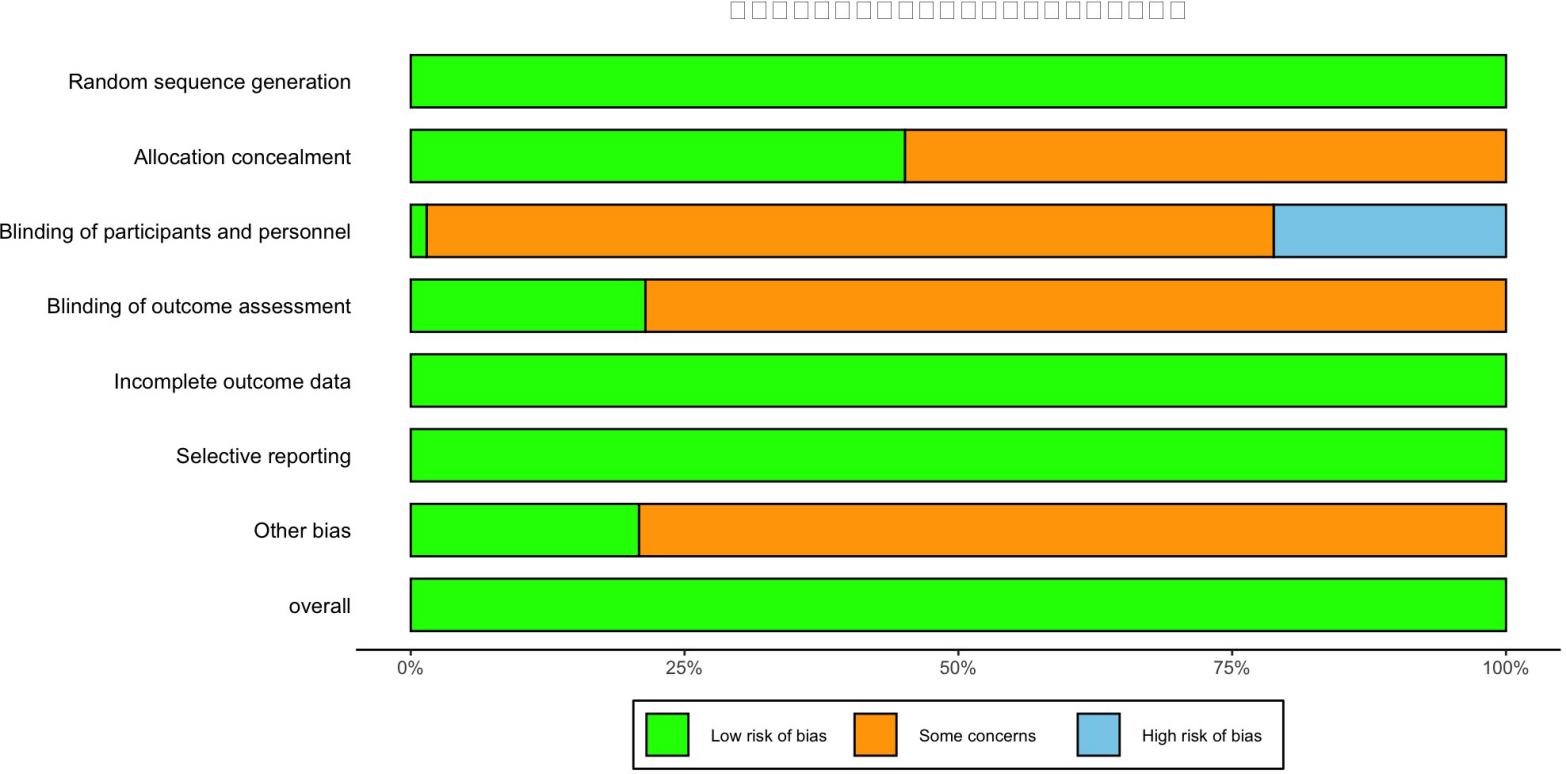
Risk of bias assessment for each RCT.

According to the study by Higgins and Green, two researchers assessed the risk of bias for each included study (**Figure 2**). A total of 67 articles were included in this study. All articles employed random allocation methods, provided detailed baseline characteristics of study participants, and clearly described intervention methods and assessment indicators. All studies reported information on allocation concealment.

#### 3.1.1 Motor abilities

motor performance was assessed using four measures (UPDRS-III, TUG test, BBS, and 6MWT). Among the 44 included studies assessing motor performance via UPDRS-III, all direct and indirect comparisons underwent consistency and inconsistency testing. With 15 total comparisons, 1 comparison showed significant inconsistency (6.7% inconsistency rate), specifically between CON and TT. Although minor inconsistencies existed within the network, overall consistency was acceptable, indicating good network consistency.

**Figure 3:**
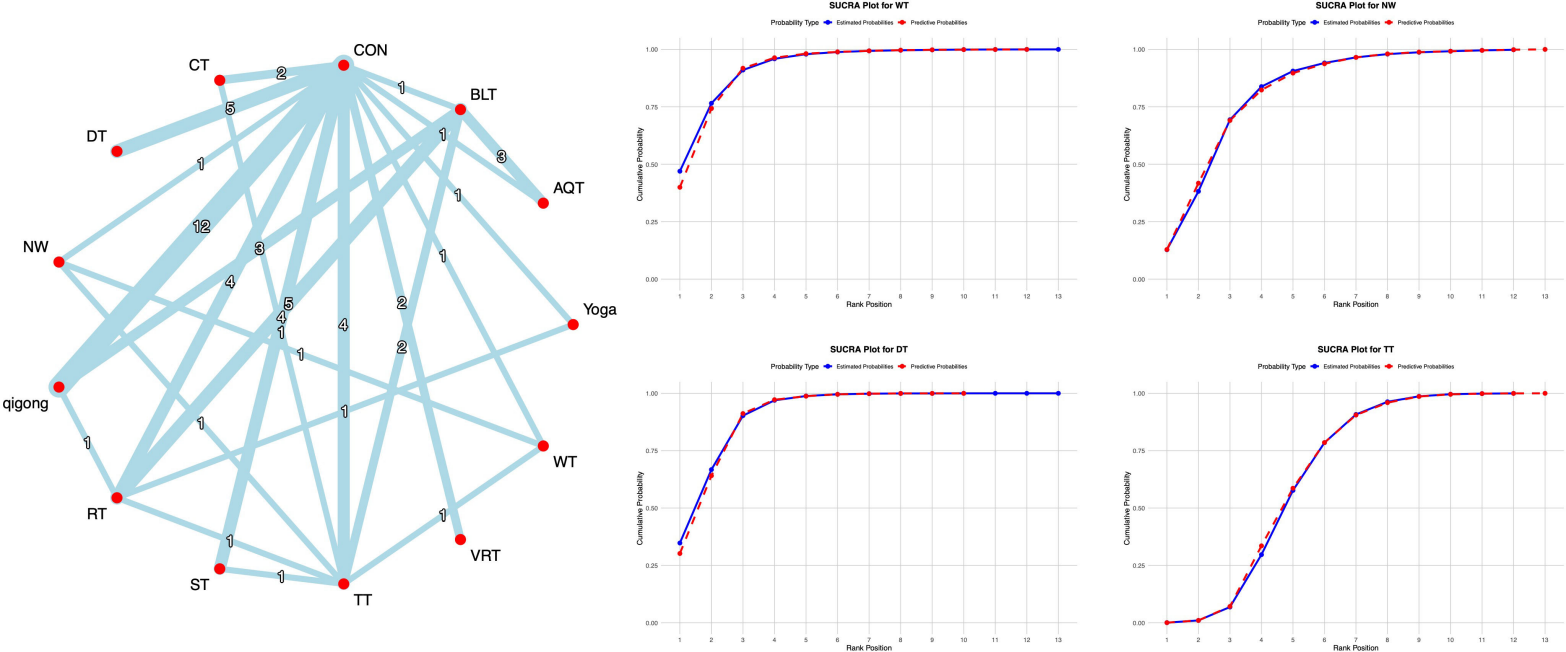
**A**-NMA figure for UPDRS-III. **B**-SUCRA plot for UPDRS-III (Top four).

Network meta-analysis results indicated that compared with conventional interventions in the control group, WT [MD = -8.46, 95% CI = (-13.62, -3.13)], DT [MD = -7.96, 95% CI = (-11.62, -4.26)], NW [MD = -6.41, 95% CI = (-11.62, -4.26)], and TT [MD = -3.31, 95% CI = (-6.14, -0.52)] were significantly lower than the control group in reducing UPDRS-III scores, as detailed in **Supplementary Table 1**. With regard to the UPDRS-III score, previous studies have shown that the MCID range for PD typically falls between 2 and 5 points (Mishra et al., 2024). In this study, all of the aforementioned exercise interventions achieved the established MCID threshold for the UPDRS-III, suggesting that these interventions not only demonstrated statistical significance but also resulted in clinically perceptible improvements in motor function for patients. Probability-based ranking of different exercise interventions showed WT ranked highest in SUCRA analysis (92.5%), followed by DT (91.2%), as illustrated in **Figure 3**.

The 34 included studies evaluated the TUG. All direct and indirect comparisons underwent consistency and inconsistency tests, yielding a total of 12 comparisons. One comparison showed significant inconsistency (8.3% inconsistency rate), specifically the CON vs RT comparison. Although minor inconsistencies existed within the network, overall consistency was acceptable, indicating good network consistency.

**Figure 4:**
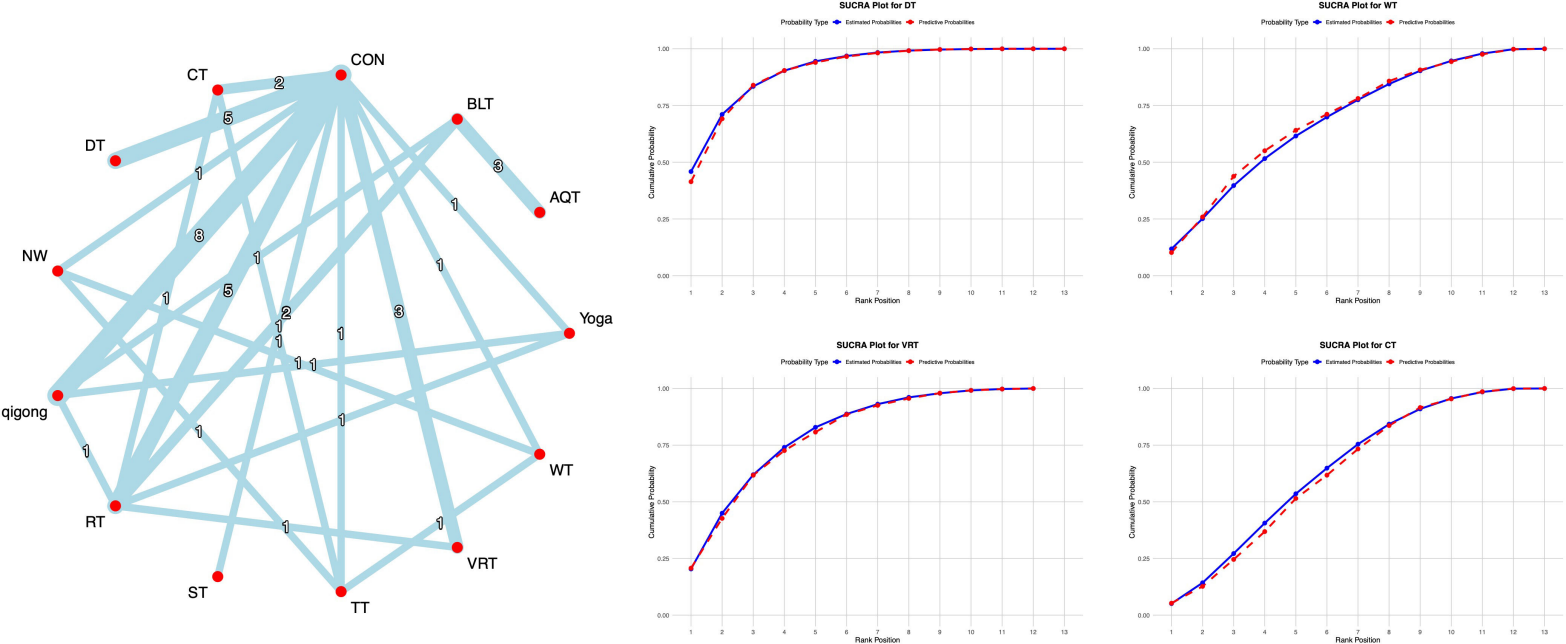
**A**-NMA figure for TUG. **B**-SUCRA plot for TUG (Top four).

The network meta-analysis results indicated that compared with conventional interventions in the control group, DT [MD = -3.11, 95% CI = (-4.91, -1.30)], VRT [MD = -2.48, 95% CI = (-4.53, -0.43)], and qigong [MD = -1.54, 95% CI = (-3.08, -0.03)] significantly reduced TUG scores compared to the control group. See **Supplementary Table 2** for details. With regard to TUG scores, previous studies have shown that the MCID range for PD is typically 1.7 seconds or more (Mishra et al., 2024). In this study, DT and VRT achieved an improvement in TUG scores that met the established MCID threshold, indicating that this intervention can provide patients with clinically meaningful improvements in motor function. Probabilistic ranking of different exercise interventions showed DT ranked first in SUCRA analysis (89.9%), as illustrated in **Figure 4**.

The 25 included studies evaluated BBS. All studies underwent consistency and inconsistency tests for direct versus indirect comparisons, with all p-values exceeding 0.05. This indicates acceptable overall consistency among studies and good network consistency.

**Figure 5:**
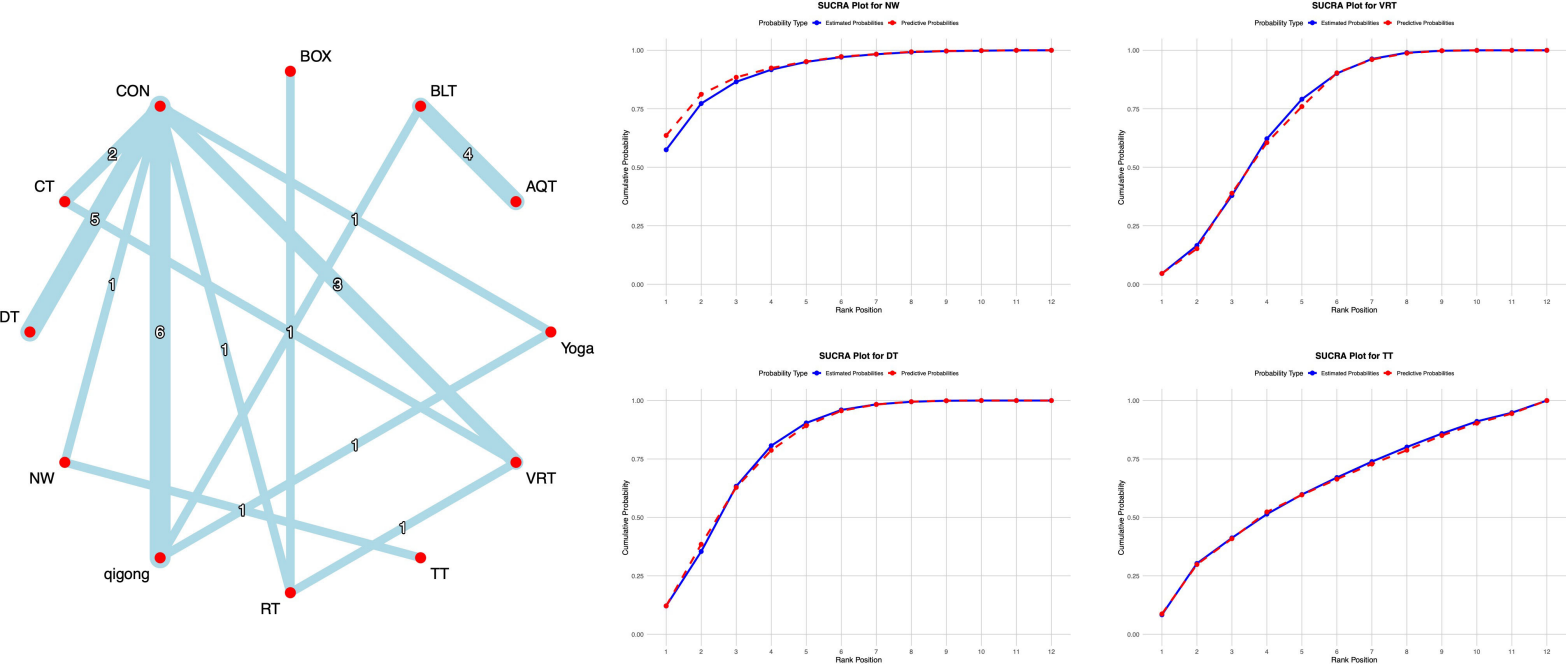
**A**-NMA figure for BBS. **B**-SUCRA plot for BBS (Top four).

The network meta-analysis results showed that compared with conventional interventions in the control group, NW [MD = 8.43, 95% CI = (2.98, 13.80)], DT [MD = 6.25, 95% CI = (3.76, 8.75)], and VRT [MD = 5.36, 95% CI = (2.64, 8.15)], qigong [MD = 3.26, 95% CI = (0.33, 6.19)] were significantly lower than the control group in improving BBS scores, as detailed in **Supplementary Table 3**. With regard to BBS scores, previous studies have indicated that the MCID range for PD is typically 3 points or more (Miyata et al., 2021). In this study, all four interventions achieved the established MCID threshold for BBS improvement, indicating that these interventions can produce clinically perceptible improvements in motor function for patients. Probabilistic ranking of different exercise interventions showed NW ranked first in SUCRA analysis (91.1%), as illustrated in **Figure 5**.

The 14 included studies evaluated the 6MWT. All studies underwent consistency and inconsistency tests for direct and indirect comparisons, with all P-values exceeding 0.05. This indicates acceptable overall consistency among all studies and good network consistency.

**Figure 6:**
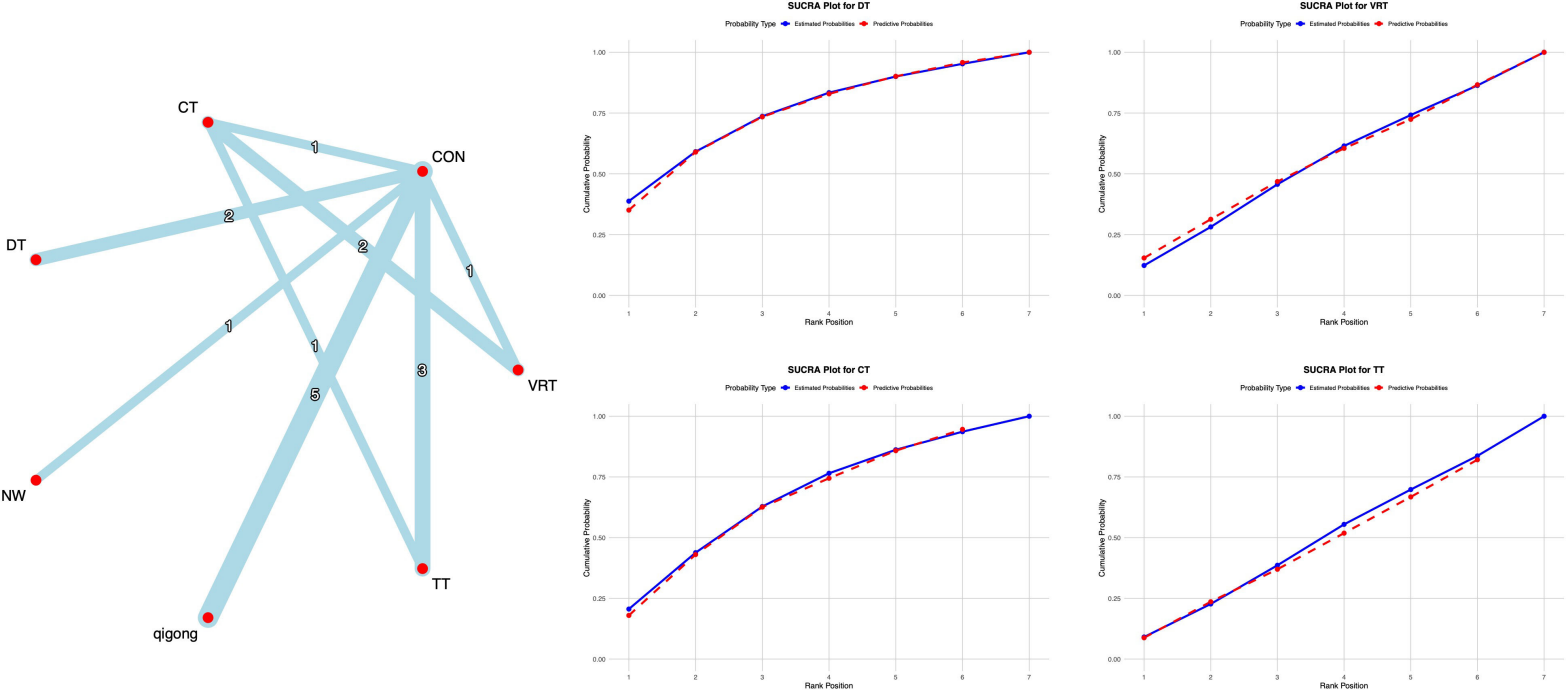
**A**-NMA figure for 6MWT. **B**-SUCRA plot for 6MWT (Top four).

The network meta-analysis results showed that compared with conventional interventions in the control group, no exercise intervention was significantly less effective than the control group in improving 6MWT scores, as detailed in **Supplementary Table 4**. Probabilistic ranking of different moderate-intensity exercise interventions revealed that DT ranked first in the SUCRA analysis (73.3%), as illustrated in **Figure 6**.

#### 3.1.2 Non-motor abilities

Non-motor abilities are primarily assessed in terms of cognitive function and quality of life. The Montreal Cognitive Assessment (MoCA) serves as a core tool for screening cognitive function in PD, and its use is supported by a solid evidence-based foundation and broad international consensus (Camargo et al., 2016). Sixteen studies employed the MoCA to assess cognitive function in Parkinson’s patients. All studies underwent consistency and inconsistency tests for direct and indirect comparison P-values, with all P-values exceeding 0.05. This indicates acceptable overall consistency among all studies and good network consistency.

**Figure 7:**
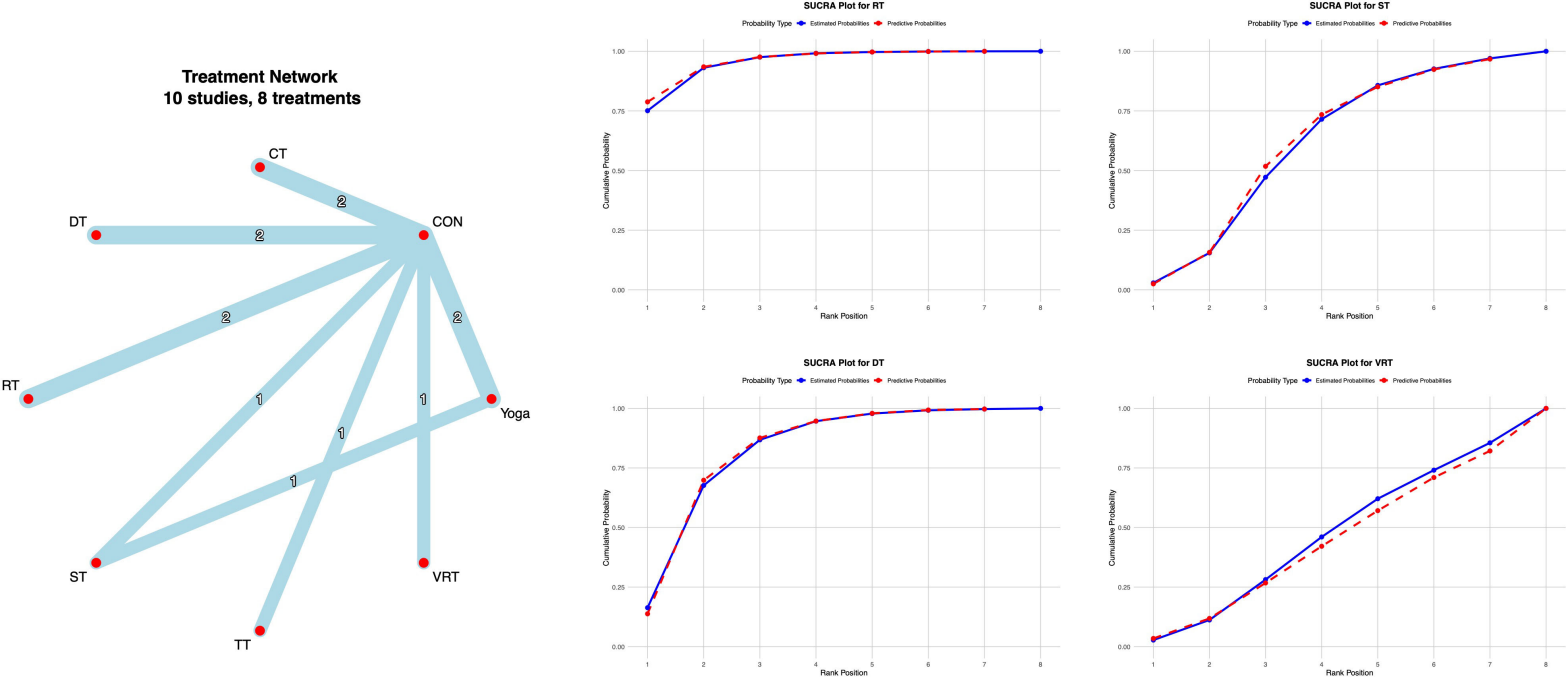
**A**-NMA figure for MoCA. **B**-SUCRA plot for MoCA (Top four).

The network meta-analysis results showed that compared with conventional interventions in the control group, RT [MD = 2.91, 95% CI = (1.28, 4.53)] and DT [MD = 1.99, 95% CI = (0.34, 3.61)] were significantly higher than the control group in improving MOCA scores, as detailed in **Supplementary Table 5**. Probability-based ranking of different moderate-intensity exercise interventions showed NW ranked first in the SUCRA analysis (91.1%), as illustrated in **Figure 7**.

Thirty-five studies used the PDQ-39 scale to assess quality of life in Parkinson’s disease patients. All studies underwent consistency and inconsistency tests for direct versus indirect comparisons, with all P-values exceeding 0.05. This indicates acceptable overall consistency among studies and good network consistency.

**Figure 8:**
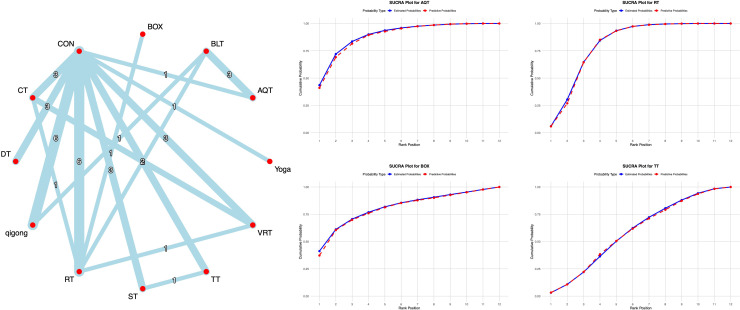
**A**-NMA figure for PDQ-39. **B**-SUCRA plot for PDQ-39 (Top four).

The network meta-analysis results showed that compared with conventional interventions in the control group, no exercise intervention was significantly less effective than the control group in improving PDQ-39 scores, as detailed in **Supplementary Table 6**. Probability-based ranking of different exercise interventions showed AQT ranked first in the SUCRA analysis (88.5%), followed by BOX (80.2%). See **Figure 8** for details.

### 3.2 Publication bias test

Funnel plots were constructed for all outcome measures to examine potential publication bias. The scatter plots exhibited broadly symmetrical distributions, indicating no significant publication bias (**Supplementary Figure 1**).

## 4 Discussion

This study systematically compared the effects of different exercise interventions on improving motor and non-motor abilities in patients with Parkinson’s disease (PD). A total of 83 exercise intervention studies were included, encompassing 11 distinct exercise modalities, with 2,642 PD patients participating—representing a substantial sample size. The results indicated that for reducing UPDRS-III scores, WT was the most effective exercise intervention, followed by DT. The optimal intervention for improving BBS scores was NW, followed by DT. DT was also the best modality for reducing TUG scores and increasing 6MWT scores. Regarding non-motor abilities, NW was the most effective intervention for improving MoCA scores, followed by DT. AQT was the best for improving PDQ-39 scores. In summary, while DT appears to be the most effective single intervention for improving motor performance based on overall analysis, NW demonstrated the highest number of indicators showing significant improvement across both motor and non-motor abilities.

Motor abilities is a critical clinical indicator for assessing recovery in PD patients. The most common symptoms of PD involve a significant decline in muscular control, including impaired balance, reduced muscular endurance, and diminished dynamic mobility (Mancini and Horak, 2016). Therefore, a comprehensive evaluation of motor performance requires multiple metrics, as relying solely on the UPDRS-III scale is often insufficient. This review employed four indicators—UPDRS-III, 6MWT, TUG test, and BBS—to assess motor performance, analyzing it from perspectives of motor impairment severity, endurance, balance, and more. For PD patients, interventions like DT, which require high neural involvement and regulation, emphasize cognitive participation and feedback mechanisms. This activates brain regions associated with movement, promotes neural plasticity, and thereby improves motor abilities (Petzinger et al., 2013). Current research suggests the primary mechanism involves enhancing neural plasticity to restore motor abilities, potentially by remodeling the morphological structure and function of dendritic spines on medium spiny neurons (MSNs) in the striatum of PD models. This may involve upregulating the expression of GluR2 subunit-containing AMPARs in the striatum, reducing calcium ion content in MSN dendritic spines, inhibiting the activity of calcium-dependent proteases and the initiation of downstream intracellular signaling cascades. Consequently, the overactivation of the striatal glutamatergic pathway is suppressed, mitigating a series of pathological changes caused by striatal overactivation in PD from the perspective of neural plasticity (VanLeeuwen et al., 2010; Toy et al., 2014; Shin et al., 2016). This leads to improved static and dynamic balance in patients, as evidenced by significant improvements in BBS and TUG scores. Furthermore, MCID threshold analysis of the TUG indicates that DT produces highly clinically significant improvements in motor function.

Furthermore, studies indicate that such exercise can also increase antioxidant capacity in PD, potentially by strengthening oxidative defense and free radical scavenging. This may prevent the release of cytochrome c from the mitochondrial respiratory chain and apoptosis-inducing factor from the mitochondrial matrix into the cytoplasm, inhibiting the initiation of apoptotic cascades and the expression of inflammatory genes. This curbs inflammatory damage to dopaminergic neurons in the substantia nigra, thereby ameliorating motor dysfunction in PD (Tajiri et al., 2010). Therefore, exercise can indeed significantly improve the motor performance of PD patients across multiple dimensions, and comprehensive analysis suggests DT is the most effective modality for this purpose.

Non-motor abilities is also a major focus in contemporary PD research. Cognitive impairment, as one of the common non-motor symptoms (Goldman and Postuma, 2014), not only severely impacts patients’ quality of life but also imposes a heavy burden on families and society. This review used the MoCA scale to assess cognitive function, and the results showed that DT could also significantly improve cognitive function in PD patients. Several studies have focused on the benefits of aerobic exercise (AE) for improving cognition in PD (Johansson et al., 2022). However, a meta-analysis indicated that while AE showed a trend toward improving cognitive function in PD patients, the difference was not statistically significant (Tang et al., 2023). This may be because cognitive dysfunction is an independent risk factor in PD, and the cognitive benefits of exercise might not be sufficient to counteract the progression of the disease itself (van der Kolk et al., 2019). Additionally, there may be a ceiling effect for aerobic exercise on cognitive improvement, making it suboptimal for addressing PD-related cognitive decline. In contrast, DT promotes neural plasticity and improves functional connectivity in the brain, particularly yielding positive effects on executive function and attention (Combs et al., 2011; Corcos et al., 2012), thereby enhancing cognitive performance.

The PDQ-39, extensively validated, is commonly used to assess patients’ health-related quality of life (Martinez-Martin and Kurtis, 2012). Mitigating the negative impact on health-related quality of life is a core objective in the management of Parkinson’s disease. This study indicates that no single form of exercise can significantly improve patients’ quality of life, which is related to the complexity and multidimensional nature of quality-of-life assessment in Parkinson’s disease. The PDQ-39 covers eight dimensions: mobility, activities of daily living, emotional state, stigma, social support, cognitive function, communication, and physical discomfort (Jones et al., 2014). Any single form of exercise may only have a significant effect on specific dimensions, while its impact on other dimensions may be less pronounced, resulting in statistically non-significant improvements in overall quality of life—a finding highly consistent with the analysis in this paper (Lacy et al., 2023). Therefore, it is particularly important to explore specialized, multidimensional exercise interventions in the future to improve patients’ quality of life.

In summary, DT appears to be an excellent option for improving patients’ motor and cognitive abilities. Furthermore, walking-based exercises (WT and NW) also achieved the recognized MCID thresholds for overall motor function, balance, and cognition. This suggests that the aforementioned exercise interventions not only yield statistically significant differences but also provide patients with clinically perceptible improvements in motor function. Therefore, future exercise programs can be tailored to patients’ specific disease stages and physical capabilities by employing different exercise modalities. This approach allows patients to transition smoothly and rapidly into the exercise phase, preventing exercise resistance in some patients who may be intimidated by the high intensity of DT. Furthermore, for patients lacking lower-limb mobility, modified forms of exercise—such as wheelchair dancing—can be adopted to better serve the needs of PD patients.

## 5 Strengths and limitations

This article incorporated a substantial number of RCTs, resulting in a large sample size that provides more objective, reliable, and credible data compared to case studies. It is the first to include traditional Chinese exercises for comparison with other exercise types, offering a more objective and comprehensive overview of exercise modalities. Furthermore, this review employed meta-analysis, which not only enhanced the statistical power of the findings but also yielded more precise data. It also helped clarify the reasons for contradictory results across different studies, providing novel and comprehensive evidence-based data.

However, this study has certain limitations. The intervention duration and frequency differed across the various exercise protocols included, with a wide range across studies. Moreover, the dosage of exercise was not analyzed, which may introduce limitations. Some of the included literature did not report the disease stage/severity of PD patients, making it impossible to determine whether the exercise interventions were effective only for those with mild symptoms. The evidence for certain exercise interventions is limited, resulting in a lack of direct comparative data for some outcomes. Therefore, the interpretation of these results should be approached with caution, and future research should aim to increase the number of studies in these areas.

## 6 Conclusion

Current evidence suggests that exercise involving walking and dance, which stimulates neural regulation, can help improve motor function and cognitive abilities in people with Parkinson’s disease. Patients can choose exercises based on their current fitness level.

## Data Availability

The original contributions presented in the study are included in the article/**Supplementary Material**. Further inquiries can be directed to the corresponding author.
