# Supplementary figures and images for: Effects of different exercises on motor and non-motor abilities in patients with Parkinson disease—a network meta-analysis of randomized controlled trials
Source: Front Physiol. 2026 May 1;17:1809614. doi: 10.3389/fphys.2026.1809614 (PMC13175863; doi:10.3389/fphys.2026.1809614)

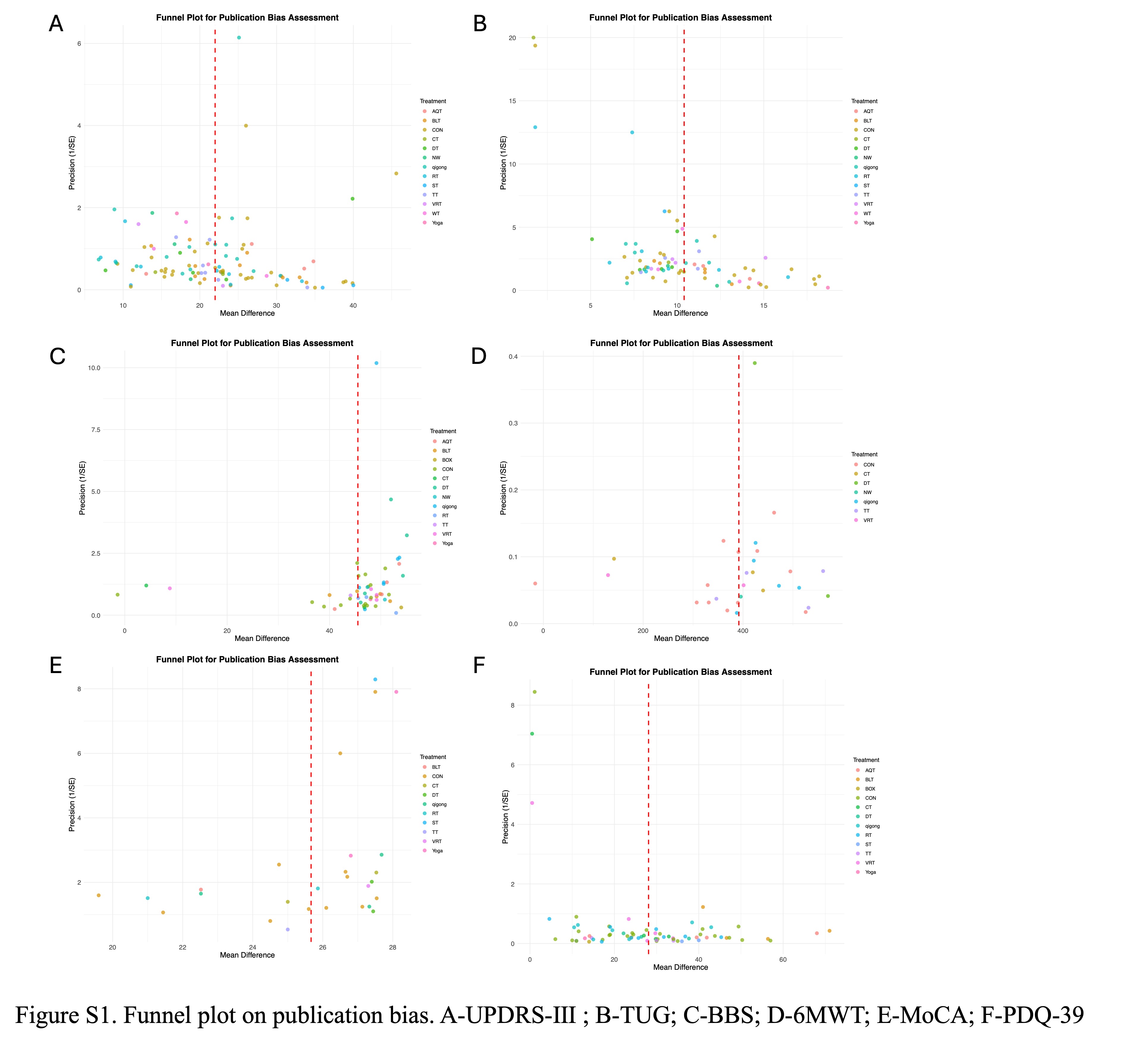

Supplement: Supplementary file 7 [file Image1.jpeg]
